# Cooling effect of urban green spaces: LCZ-based assessment comparing four cities at similar latitudes via hotspot and regression models

**DOI:** 10.1007/s00484-026-03172-x

**Published:** 2026-03-19

**Authors:** Dilara Yilmaz, Oznur Isinkaralar, Kaan Isinkaralar, Emmanuel Yeboah, Isaac Sarfo, Ayyoob Sharifi, Sevgi Öztürk, Collins Oduro, Ali Soltani, Mohsen Roohani Qadikolaei

**Affiliations:** 1 Department of Landscape Architecture, Graduate School of Natural and Applied Sciences, Kastamonu University, Kastamonu, 37150 Türkiye; 2 Department of Landscape Architecture, Faculty of Engineering and Architecture, Kastamonu University, Kastamonu, 37150 Türkiye; 3 Department of Environmental Engineering, Faculty of Engineering and Architecture, Kastamonu University, Kastamonu, 37150 Türkiye; 4 School of Remote Sensing and Geomatics Engineering, Nanjing University of Information Science and Technology, Nanjing, Jiangsu 210044 China; 5 College of Geography and Environmental Science, Henan University, Kaifeng, Henan Province 475004 China; 6 The IDEC Institute and Network for Education and Research on Peace and Sustainability (NERPS), Hiroshima University, Higashi-Hiroshima, 739-8529 Japan; 7 Department of Geography and Spatial Information Techniques, Ningbo University, Ningbo, Zhejiang 315211 China; 8 City Futures Research Centre, University of New South Wales, Sydney, Australia; 9 School of Humanities, Arts and Social Sciences (HASS), University of New England, NSW, Australia; 10 Department of Urban Planning, University of Guilan, Rasht, Iran

**Keywords:** Climate action, Land surface temperature, Life on earth, Sustainable towns and cities, Urban heat island

## Abstract

Urban green spaces (UGSs) are widely recognized as a key strategy to mitigate urban heat islands (UHIs) through evapotranspiration and shading. However, their cooling effectiveness depends not only on size but also on spatial configuration and surrounding urban morphology. This study provides a cross-latitude, local climate zone (LCZ)-based comparative analysis of UGS cooling effects in four medium-sized cities located at similar latitudes but with distinct morphological patterns: Salt Lake City (USA), L’Aquila (Italy), Krakow (Poland), and Kastamonu (Türkiye). Using MODIS-derived land surface temperature (LST), LCZ classification, and Getis-Ord (Gi*) hotspot analysis, we quantified spatial clustering of heat. We identified where parks and natural landscapes are most effective in lowering temperatures. Furthermore, Ordinary Least Squares (OLS) regression was used to establish global relationships between green space coverage and LST, followed by Geographically Weighted Regression (GWR) to capture local variations and spatial heterogeneity in this relationship. Results reveal that green areas significantly lower LST, with the strongest cooling observed within 400 m of parks. Rather than treating this buffer distance as a universal planning threshold, the analysis demonstrates that cooling magnitudes and spatial consistency vary systematically across LCZ types and urban contexts. In Salt Lake City, for instance, UGSs reduced temperatures by up to 4.2 °C in compact residential zones (LCZs 2–3). Hot spot analysis further shows that statistically significant heat clusters (95–99% confidence) are concentrated in dense, built-up LCZ types, while cold spots are predominantly associated with natural LCZ classes (A–D). Together, these findings suggest that urban morphology not only influences the presence but also the effectiveness of cooling buffers around green spaces. By integrating LCZ mapping, spatial statistics, and a comparative, cross-latitude design, this study moves beyond distance-based generalisations. It advances urban climate research by demonstrating a transferable yet context-sensitive framework for diagnosing UHI intensity and prioritising green space interventions. The results provide actionable guidance for planners: park siting and design should prioritize heat-prone, high-density zones to maximize cooling benefits and support climate-resilient urban development.

**Graphical abstract:**

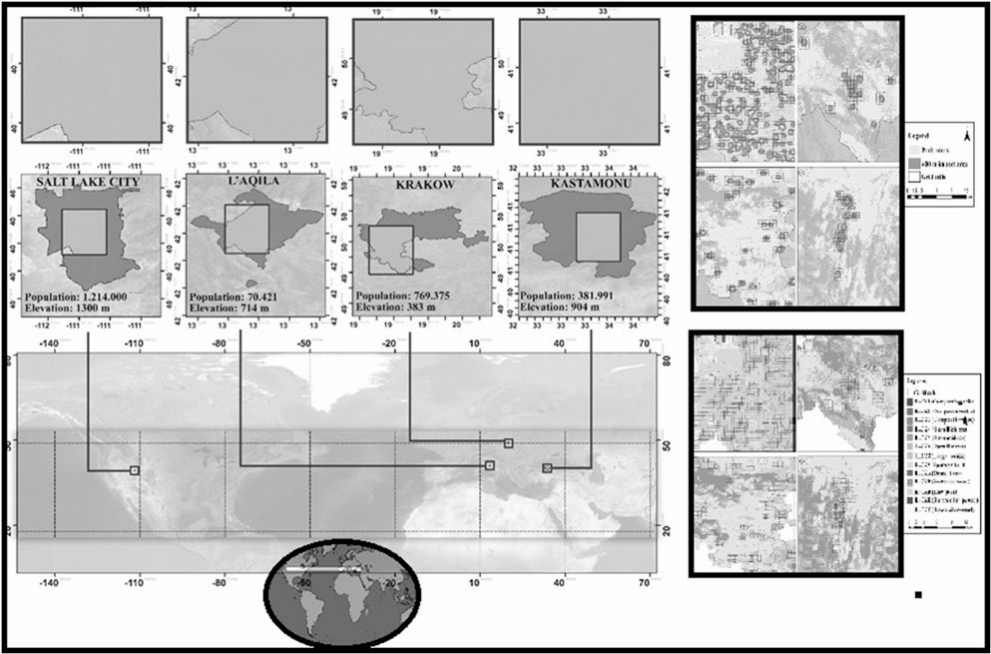

**Supplementary Information:**

The online version contains supplementary material available at 10.1007/s00484-026-03172-x.

## Highlights

The spatial distribution of the cooling effects of UGS on four cities was determined.

The relationship between UGS and LST was evaluated comparatively using LCZ.

The cooling effects of UGS were compared among LCZs.

Getis-Ord Gi* analysis revealed significant heat/cool clusters, indicating priority zones for green infrastructure.

The strongest cooling was observed within 400 m of parks, particularly in compact, dense LCZs.

**Supplementary Information:**

The online version contains supplementary material available at 10.1007/s00484-026-03172-x.

## Introduction

Urbanization has emerged as one of the most transformative human processes, reshaping landscapes and altering local climates worldwide (Taubenböck et al. 2024; Krüger et al. 2024). The rapid expansion of urban areas has increased the urban heat island (UHI) effect. In this phenomenon, cities experience significantly higher temperatures than surrounding rural areas due to multiple factors (Zezzo et al. 2025). As global temperatures rise due to climate change, the need for effective urban heat mitigation strategies has become increasingly urgent (Abubakar et al. 2025). Urban Green Spaces (UGSs), including parks, street trees, and green roofs, have been widely recognized as nature-based solutions to counteract the UHI effect (Aram et al. 2019; Iungman et al. 2025). These solutions provide cooling through multiple mechanisms: vegetation shades surfaces, reducing solar radiation absorption; evapotranspiration converts solar energy into latent heat, lowering ambient temperatures; and green infrastructure modifies surface albedo, enhancing heat dissipation (Zölch et al. 2016). However, the magnitude and spatial extent of these cooling effects are strongly scale-dependent and context-specific, varying across neighborhood, city, and regional scales. The cooling capacity of UGS is therefore not uniform; it varies substantially depending on factors such as vegetation type, density, spatial configuration, and the surrounding urban fabric. Understanding these variations at an appropriate spatial scale is critical for optimizing green space design and placement to maximize thermal benefits.

The local climate zone (LCZ) classification system developed by Stewart and Oke (2012) offers a standardized framework to investigate these dynamics by categorizing urban and natural landscapes based on their morphological and land cover properties (e.g., building height, impervious surface fraction, vegetation cover). LCZs (λs) enable comparisons of thermal environments between cities with different forms and densities (Ghanbari et al. 2023; RoohaniQadikolaei et al. 2025). Although several studies have explored the relationships between λ types and land surface temperature (LST) (Yang et al. 2021; Rosier et al. 2025), most have focused on single cities or cities located at different latitudes and under varying climatic regimes. Moreover, evidence derived from coarse-resolution satellite products may lead to generalized interpretations if scale effects are not explicitly acknowledged. Comparative LCZ-based analyses of UGS cooling effects across cities with similar latitudes and climate conditions but different morphological patterns remain rare. Building on this perspective, the present study focuses on cities with similar latitudinal positions but contrasting morphological features. Rather than generalizing cooling performance, the study aims to interpret UGS effects within the spatial and methodological constraints of the applied datasets.

This study addresses this gap by conducting a cross-country, LCZ-based comparative analysis of UGS cooling effects in four mid-latitude cities located at similar latitudes but with distinct urban forms: Salt Lake City (USA), L’Aquila (Italy), Krakow (Poland), and Kastamonu (Türkiye). These cities were strategically selected to control for latitude and coastal influences while representing diverse population densities, green space distributions, and built morphologies (Isinkaralar et al. 2026). The analysis is conducted at the city and intra-urban scale using MODIS-derived LST data, which allows for consistent cross-city comparison but inherently reflects neighborhood-scale thermal patterns rather than micro-scale temperature variations. Using MODIS-derived LST, LCZ mapping, and Getis-Ord Gi* hot spot analysis, this study identifies spatial clusters of heat and examines where parks and natural landscapes most effectively mitigate temperature increases. Furthermore, Ordinary Least Squares (OLS) regression is used to assess global, city-wide relationships between green space coverage and λ, followed by Geographically Weighted Regression (GWR) to capture localized spatial heterogeneity in these relationships across λ types. This multi-method approach allows the study to disentangle the roles of local morphology and green space structure from broader climatic factors (Isinkaralar et al. 2025a, b). By explicitly documenting the analytical scale and methodological choices, the study aims to avoid overinterpreting statistical associations as direct causal effects. In this context, study questions are designed as follows:

- SQ1: How do UGS impact LST across different LCZs?
- SQ2: Do the cooling effects of UGS vary significantly across LCZ types?
- SQ3: Are UGS equally effective in mitigating LST in cities with similar latitudes and morphological features?
- SQ4: How are statistically significant hot and cold spots of LST distributed across LCZ types, and to what extent do they spatially overlap with UGS?

By explicitly testing the consistency of UGS cooling effects across comparable λs in different national contexts, this study contributes new evidence to urban climate research. Rather than proposing universal cooling thresholds, the findings highlight relative differences in UGS performance across λs and cities. This evidence-based framework can inform spatial planning policies by linking local morphological characteristics with heat mitigation potential. For urban planners, the results provide practical guidance on where green space interventions are likely to be most effective at the neighbourhood and district scale, supporting location-specific strategies rather than one-size-fits-all solutions. The findings can support planners and policymakers in developing targeted, location-specific green infrastructure strategies to mitigate urban heat in the most vulnerable and densely built areas.

## Material and method

### Study area

**Fig. 1:**
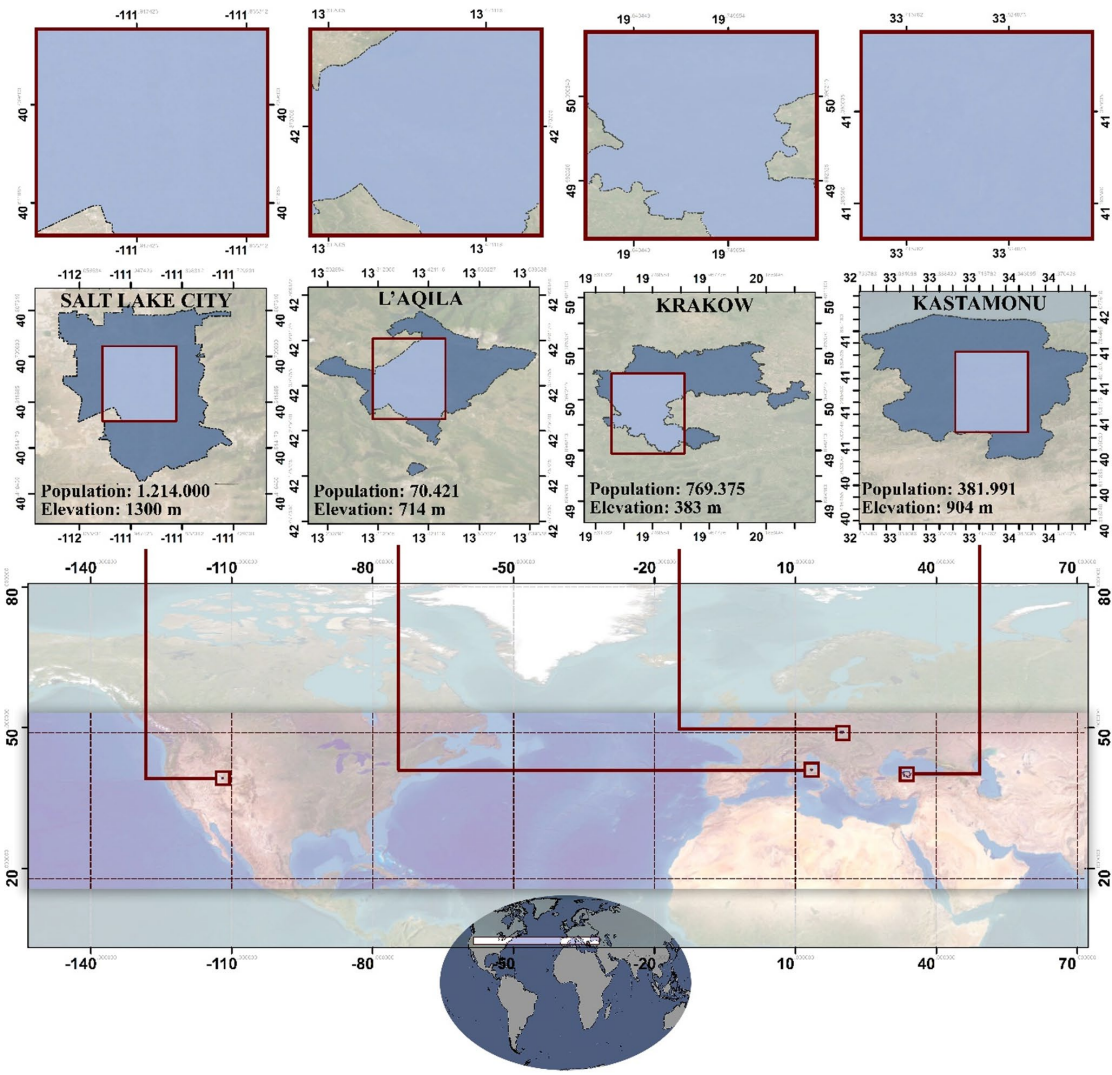
Locations of the selected cities

In the study aiming to determine the cooling effects of UGSs in cities with similar morphological features, *“the sample area selection”* was completed in 3 stages. (1) First, medium-sized cities at the same latitude, far from the influence of external dynamics, were determined. (2) Cities with coastal effects were eliminated. (3) After latitude, scale, and maritime criteria, population size, elevation, and settlement areas are the effective criteria for selection. In this context, residential areas in four different cities were investigated: Salt Lake City in the state of Utah, USA, L’Aquila in the Abruzzo Region of Italy, Krakow in the Malopolska Voivodeship (Lesser Poland Region) of Poland, and Kastamonu in the Black Sea Region of Türkiye, as shown in Fig. 1.

**Fig. 2:**
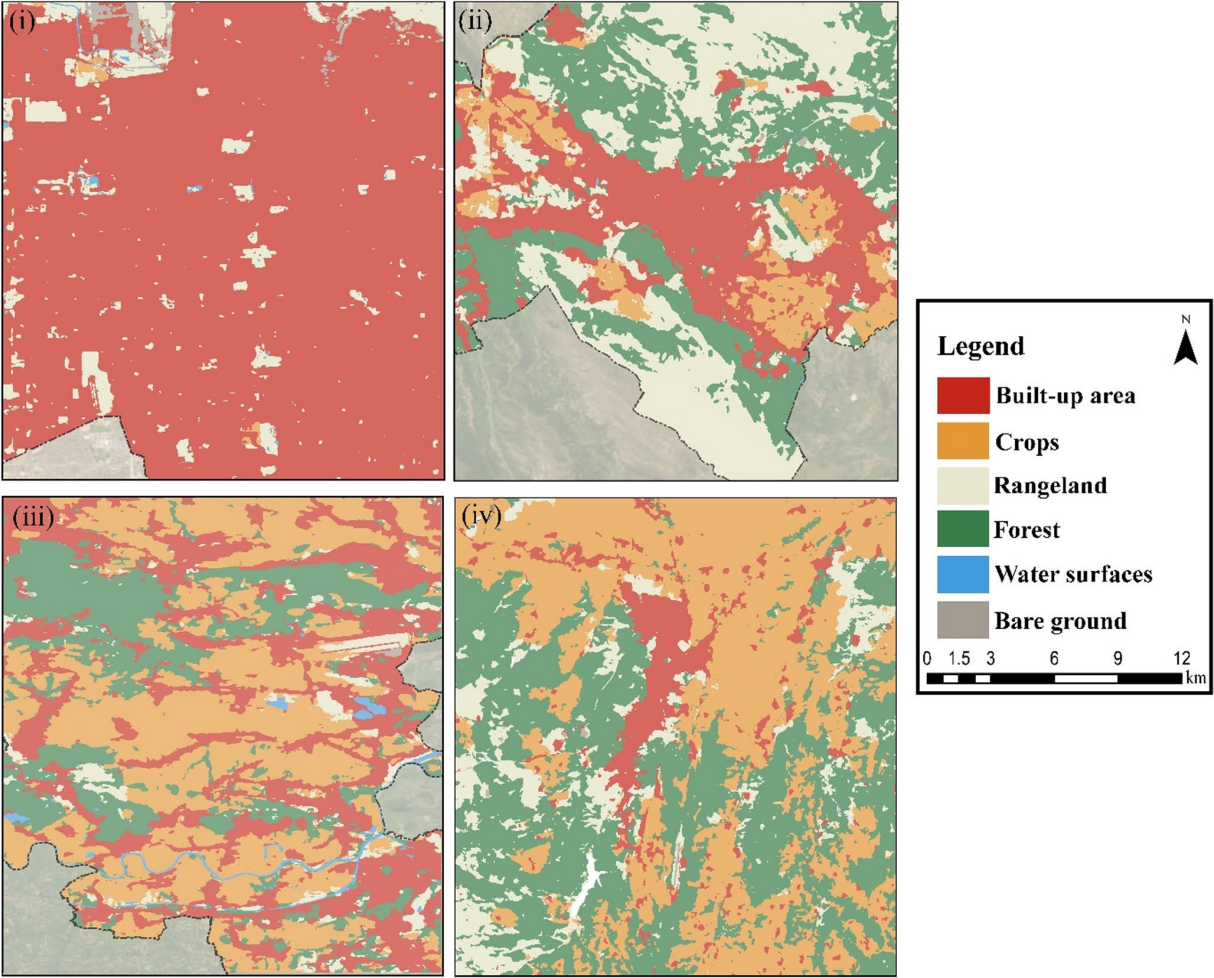
LULC maps (i: Salt Lake City, ii: L’Aquila, iii: Krakow, iv: Kastamonu)

The main selection criteria are that the majority of the sample areas are residential areas and also a central settlement unit (Appendix Table S1). When the spatial distribution of land cover/land use (LULC), which is effective in the selection of the sampling area, is examined, it is seen that approximately 85% of Salt Lake City consists of dense and large built areas approximately 27% of L’Aquila consists of scattered and less dense built areas, approximately 30% of Krakow consists of built areas intertwined with crops areas, and approximately 16% of Kastamonu consists of built areas within dense forests and crops areas (Fig. 2). These LULC maps are provided to illustrate the general landscape characteristics of the study areas and to highlight the environmental and morphological diversity considered during the sample selection process. It is worth noting that the analytical procedures in this study were conducted using grid cells, as detailed in the Methodology section. The maps shown here are for contextual purposes only and do not reflect the spatial units used in the statistical analyses.

### Methodology

The study investigates the relationship between UGS and LST at the grid-cell scale. The methodology consisted of three main stages. First, LST and local λ maps of the study area were generated. Second, park areas within the urban settlements were identified and delineated. To enable spatially explicit analysis, the study area was divided into grid cells of 500 *500 m. This resolution was chosen as a balance between spatial detail and statistical robustness: finer grids (e.g., < 250 m) would fragment large parks across multiple cells, biasing the estimation of cooling effects, while coarser grids (> 1 km) would mask local λ heterogeneity and reduce the ability to detect park-related cooling. The selected resolution captures the dominant land cover and λ composition of each neighborhood-scale unit while maintaining computational feasibility for multi-city comparison. Similar scales (250–500 m) have been recommended for neighborhood-scale UHI assessments in previous research (Sinsel et al. 2021; Ouyang et al. 2022; Kotharkar and Dongarsane 2024; Wang et al. 2024).

Although Moderate Resolution Imaging Spectroradiometer (MODIS) LST data have a native spatial resolution of approximately 1 km, the analysis was conducted using a 500 m grid framework to enable spatial integration with λ maps and UGS layers.

MODIS LST values were not spatially downscaled to infer sub-pixel temperature variability; instead, each 500 m grid cell was assigned LST values through spatial aggregation and resampling procedures that preserve the original MODIS signal. This approach allows consistent spatial alignment across datasets, while avoiding artificial enhancement of spatial detail beyond the sensor’s intrinsic resolution. The use of a 500 m grid, therefore, serves as an analytical framework, rather than a representation of micro-scale thermal heterogeneity.

Consequently, the results capture neighbourhood- and district-scale thermal patterns associated with λs and green space distribution, rather than fine-scale microclimatic variations, such as street-level shading effects.

**Fig. 3:**
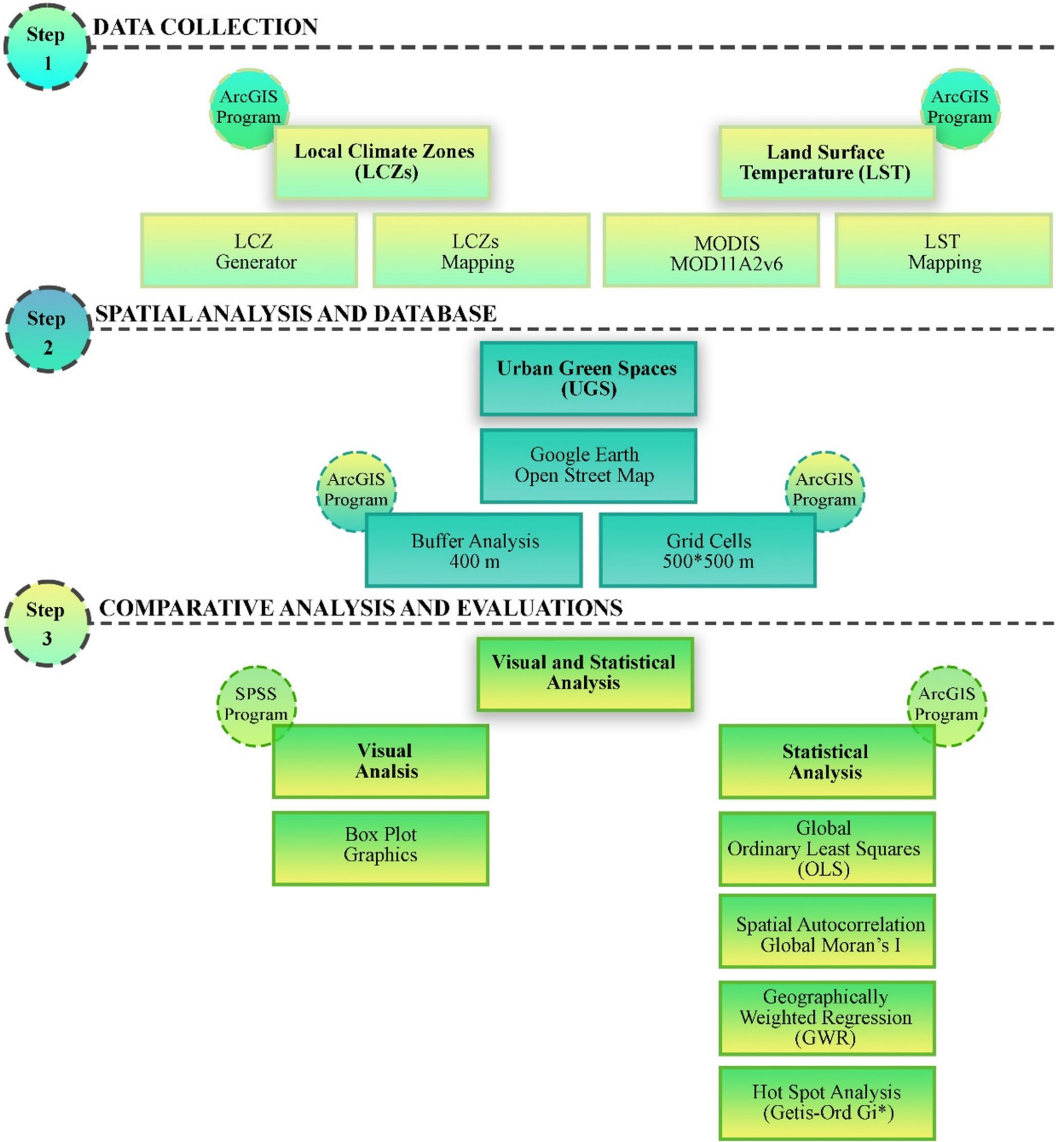
Workflow designed for this study

Cells located within 400 m of park areas were selected to represent the potential cooling influence. This buffer distance aligns with prior research, which indicates that the cooling effect of UGS is most pronounced within 400–500 m (Masoudi et al. 2019; Qiu and Jia 2020; Lin et al. 2023; Kirschner et al. 2023). Shorter distances are often preferred because they better capture microclimatic impacts and reduce noise from broader-scale climatic variation. In urban climate studies using grid-based analyses, MODIS or Landsat LST maps are commonly aggregated to grid units (e.g., 100 m, 500 m) to facilitate spatial modeling and interpretation (Crosson et al. 2012; Yang et al. 2021; Jeon et al. 2023; Chen et al. 2023). A 400 m buffer distance was applied consistently across all four cities to examine the spatial extent of the cooling influence of UGS within a comparable analytical framework (Fig. 3). This distance was not defined as a universal or physically fixed threshold at which cooling effects cease; rather, it represents a reference distance commonly used in urban climate literature to capture the near-field influence of green spaces at the neighbourhood scale. Given the pronounced differences in urban morphology, density, and green space configuration among Salt Lake City, L’Aquila, Kraków, and Kastamonu, the spatial reach of cooling effects is expected to vary across cities. The use of a uniform buffer distance, therefore, enables cross-city comparison of relative cooling gradients while acknowledging that the absolute magnitude and decay rate of cooling may differ depending on local morphological characteristics.

In this context, the identified “turning point” around 400 m should be interpreted as an analytical indicator of diminishing cooling influence under the applied spatial and climatic conditions, rather than as a universally applicable cooling boundary. This approach allows the study to highlight how urban form and green space structure modulate cooling effectiveness across different cities, supporting morphology-sensitive interpretations rather than generalized thresholds.

Finally, the effect of park areas on LST within λ units was evaluated using statistical analyses, allowing a comparative assessment of cooling effects across different λ types.

#### Classification of LCZs (λs)

The World Urban Database and Access Portal Tools (WUDAPT) λ procedure was applied to determine the λ classes for the study area. The procedure is based on the global λ raster, as defined and accessible using the WGS system by Demuzere et al. (2022).

#### Retrieval of LST from the MODIS data

The study utilized the MOD11A2v6 product (1 km spatial resolution) from the Terra MODIS collection to determine LST in summer 2023. MODIS LST data were obtained from the NASA database (https://urs.earthdata.nasa.gov/home). MODIS data were converted from MODIS units to LST in degrees Celsius by applying the MODIS scale factor. In calculating the LST of the study area, 3-month average values were determined for June, July, and August.

Equation 1 was used in the calculation of MODIS LST:

1 $$LST_{\left({}^\circ C\right)}=\left(MD\times SF\right)-273.15$$

Where LST (°C): is the LST in Celsius. MD: MODIS data. SF: MOD11A2 represents the satellite image scale factor (0.02).

MODIS-derived LST data were used to capture seasonal thermal patterns during the summer months (June–August 2023). While finer-resolution sensors, such as Landsat, can better represent micro-scale variability, MODIS was selected due to its consistent multi-temporal coverage, which enables the calculation of three-month averaged LST values and minimizes data gaps caused by cloud contamination (Appendix Table S2). This trade-off between spatial resolution and temporal consistency is particularly relevant for cross-city comparative analyses, where methodological consistency across study areas is critical.

#### Urban green spaces (UGS)

**Fig. 4:**
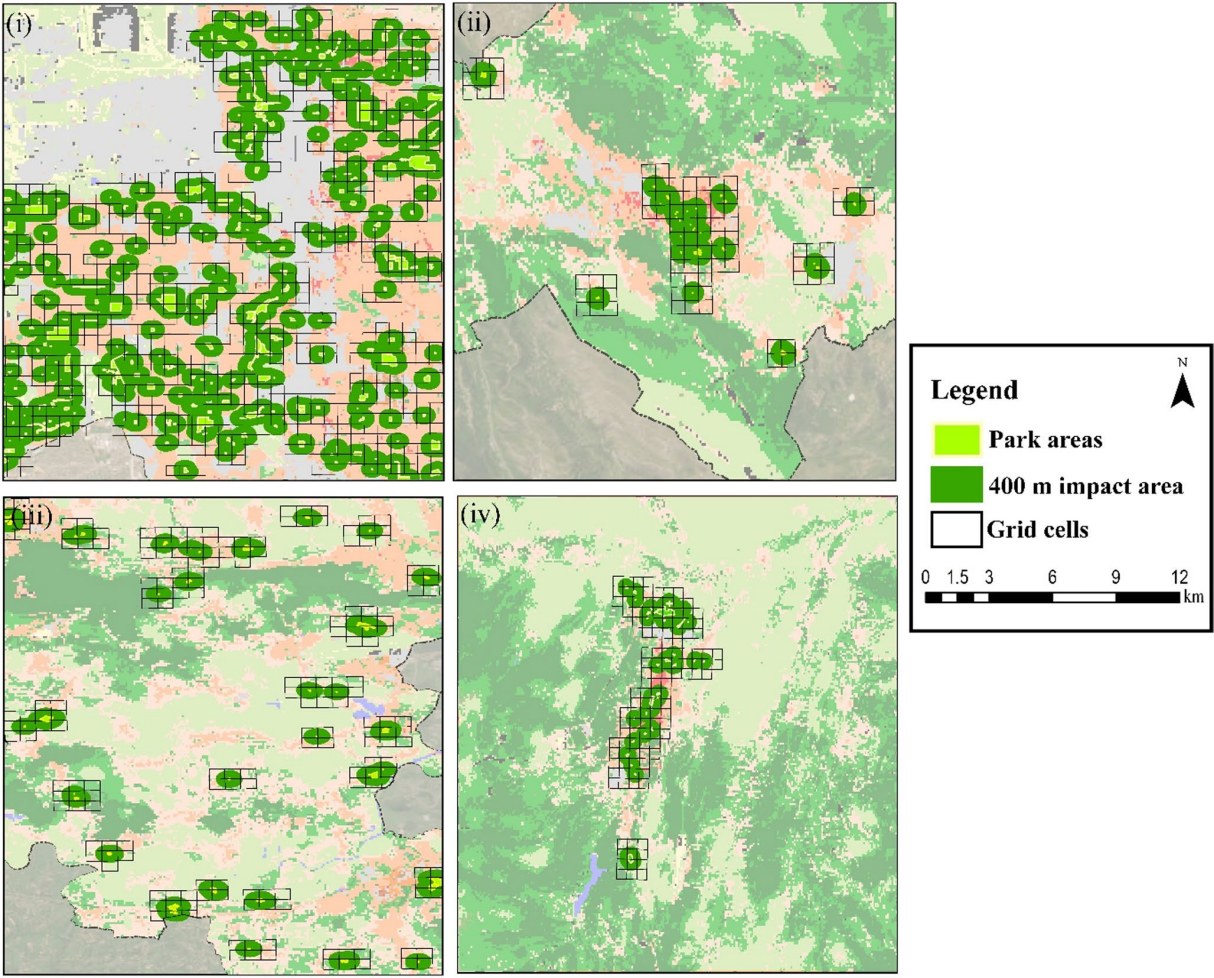
Maps of λ units within 400 m of UGS (i: Salt Lake City, ii: L’Aquila, iii: Krakow, iv: Kastamonu)

The study area UGSs were obtained from Google Earth and OpenStreetMap databases in polygon format. After the UGSs were obtained, a 400-m buffer analysis was performed on these areas within the GIS environment. After the buffer zones were determined, the λ units in these regions were identified. Salt Lake City has 308 park areas, and 967 cells were defined within the 400 m impact area. In L’Aquila, there are 46 park areas, and 89 cells were defined within the 400 m impact area. In Krakow, there are 62 park areas, and 193 cells were defined within the 400 m impact area. In Kastamonu, there are 50 park areas, and 125 cells were defined within the 400 m impact area. Comparative analyses and evaluations were made in 1374 cells (Fig. 4).

#### Measuring the relationship between urban green spaces (UGS) in LCZ units

The relationship between the average LST values of λ units with UGS was analyzed in SPSS 23 and ArcGIS 10.1.1 programs. Both visual analysis methods and statistical analysis methods were applied. Box plots were first generated in SPSS 23 to visualize the distribution of average LST values within λ units located in the 400 m impact zones around parks. For regression analyses, the dependent variable was defined as the average summer LST (June–August), while the independent variable was the λ type codes of the units within the 400 m park buffers. Global OLS regression and Geographically Weighted Regression (GWR) were then applied in ArcGIS 10.1.1 to assess both global and spatially explicit local relationships, providing a comprehensive evaluation of the cooling effects of UGS based on λ types.

#### Mapping statistically significant LST clusters

Point-based surface temperature (LST) data were analyzed using the Getis-Ord (Gi*) statistic to identify spatial clusters. The resulting vector hot spot outputs were converted to raster format using ArcGIS’s Point to Raster tool, with a cell size of 500 m and the GiZScore area as the cell value. This streamlined process enabled a direct comparison of surface temperature maps and the identification of statistically significant hot and cold spatial patterns in a consistent format.

## Results

### LST within LCZs

**Fig. 5:**
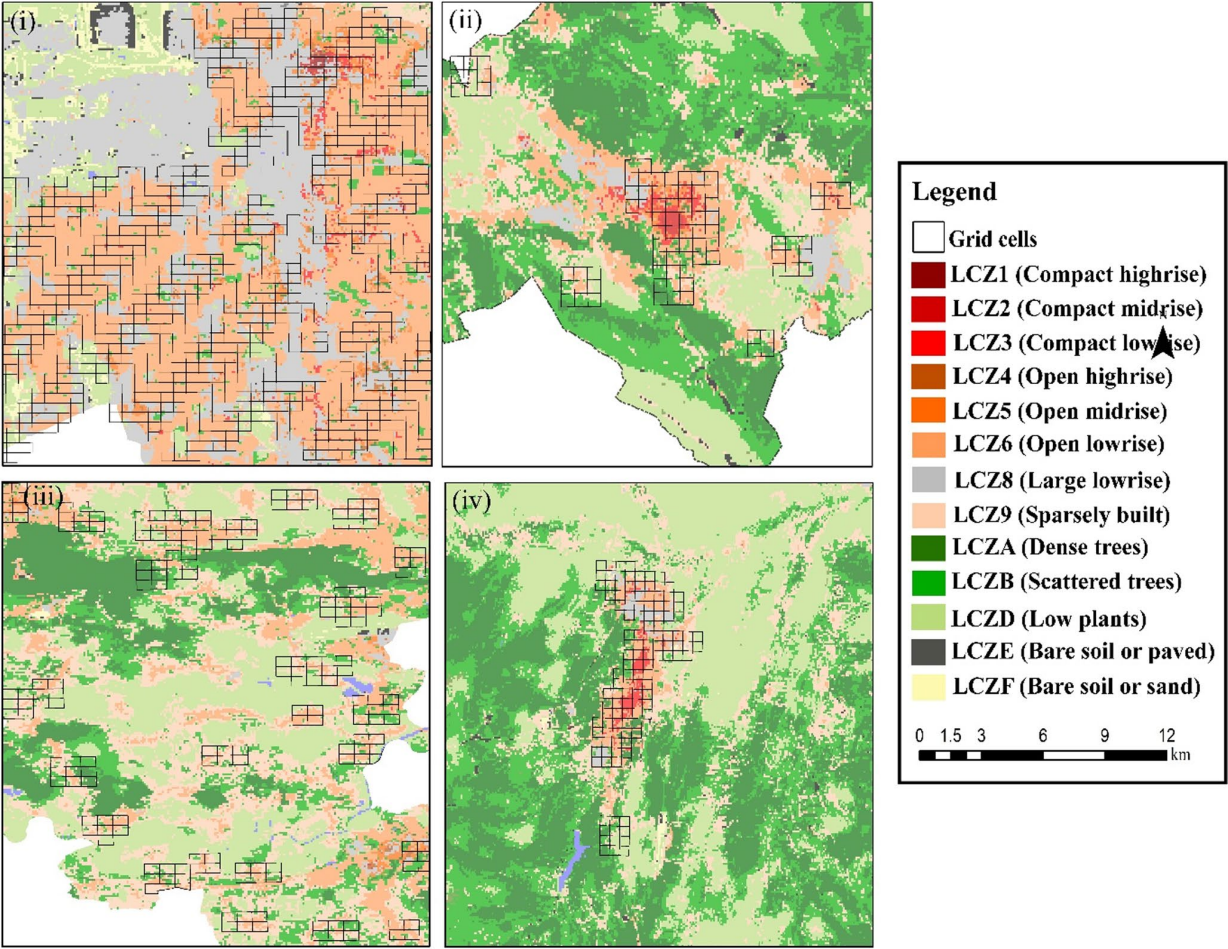
Maps of study area LCZs (i: Salt Lake City, ii: L’Aquila, iii: Krakow, iv: Kastamonu)

A comparative analysis of λ distributions across the four study cities revealed notable differences in urban morphology. Salt Lake City exhibited the most diverse set of λ types (13 in total), with urban λs (LCZ1–LCZ6, LCZ8–LCZ9) predominantly aligned along the north-south axis and natural λs (LCZA, LCZB, LCZD, LCZE, LCZF) concentrated at the periphery. In contrast, L’Aquila, Krakow, and Kastamonu each contained 9 LCZ types. In these European and Turkish cities, urban LCZs (LCZ2–LCZ6, LCZ8–LCZ9) were mainly located in the city center, while natural LCZs (LCZA, LCZB, LCZD, and in some cases LCZF) occupied larger peripheral areas. Overall, Salt Lake City demonstrates the highest λ diversity, whereas the European cities and Kastamonu are characterized by a stronger dominance of natural λ types around compact urban cores (Fig. 5).

**Fig. 6:**
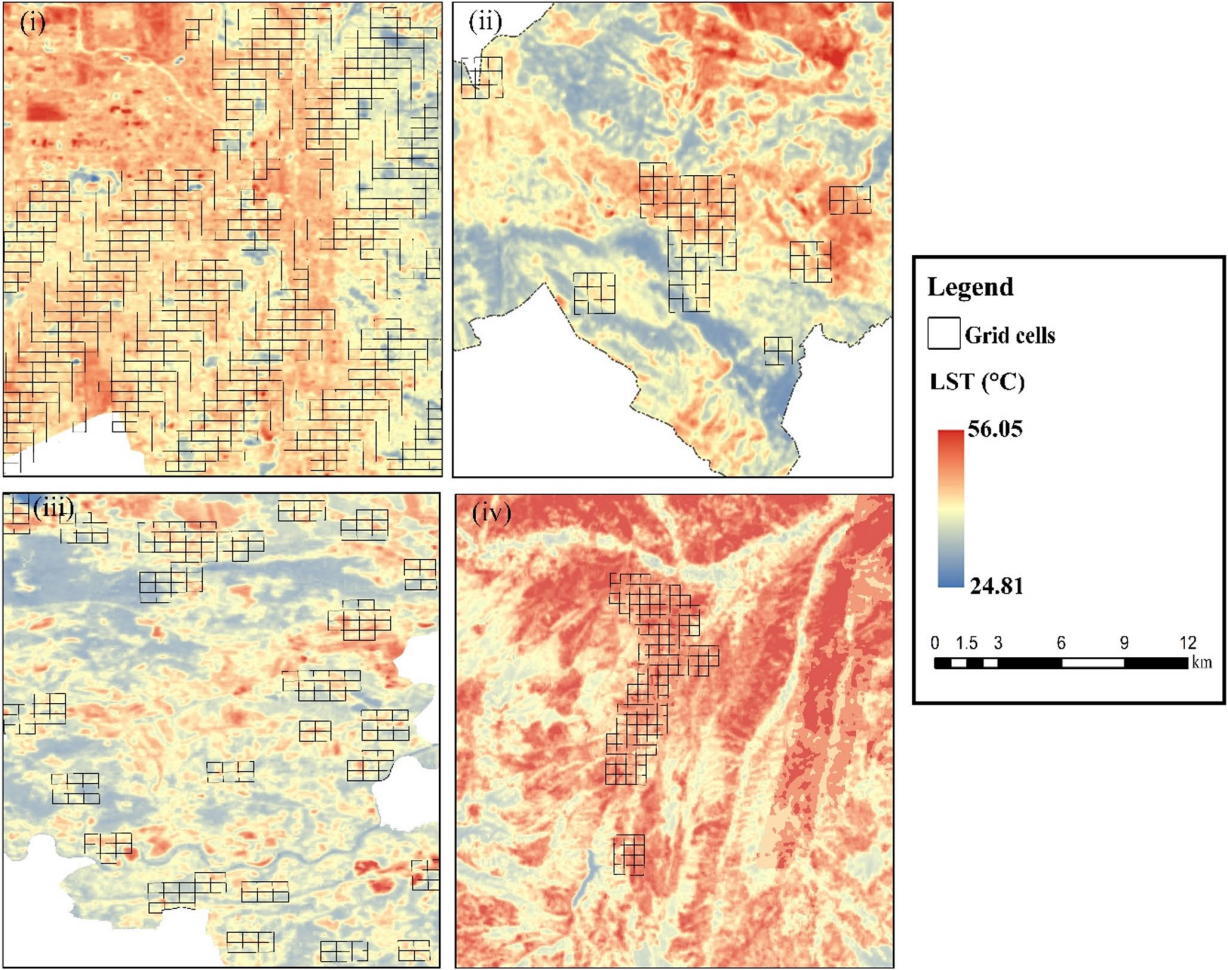
Maps of study area average daytime LST 3-month mean (i: Salt Lake City, ii: L’Aquila, iii: Krakow, iv: Kastamonu)

The daytime LST map of the study areas is provided for the summer period (June–August 2023). During this time, surface temperature values range between approximately 24.81 °C and 56.05 °C. It is observed that daytime temperature values increase in all cities, especially in areas where urban λs are concentrated. In contrast, relatively lower temperature values are observed within the 400 m impact zones of park areas. This pattern is particularly evident in Salt Lake City, Krakow, and Kastamonu (Fig. 6).

### Statistical relationship between UGS and LST in LCZs

LST values in LCZ units were calculated 400 m away from UGS. The average LST values in the study areas are presented as box plots in **Appendix Figure ****S1**. LST values are high in Salt Lake City, where urban areas are the densest. The fact that the highest median value of 56.26 °C is in Salt Lake City supports this result. Kastamonu follows Salt Lake City. High LST values are seen in Kastamonu. The median value was calculated as 55.84 °C. The median values are also lower in L’Aquila and Krakow, where LST values are relatively lower compared to other cities. While the median value in L’Aquila is 41.20 °C, it is calculated as 40.44 °C in Krakow. In all study areas, LST values in natural types are lower than LST values in built types. This is an important indicator of the reducing effect of natural areas on LST values.

### Cooling effects of parks identified through hot spot analysis

**Fig. 7:**
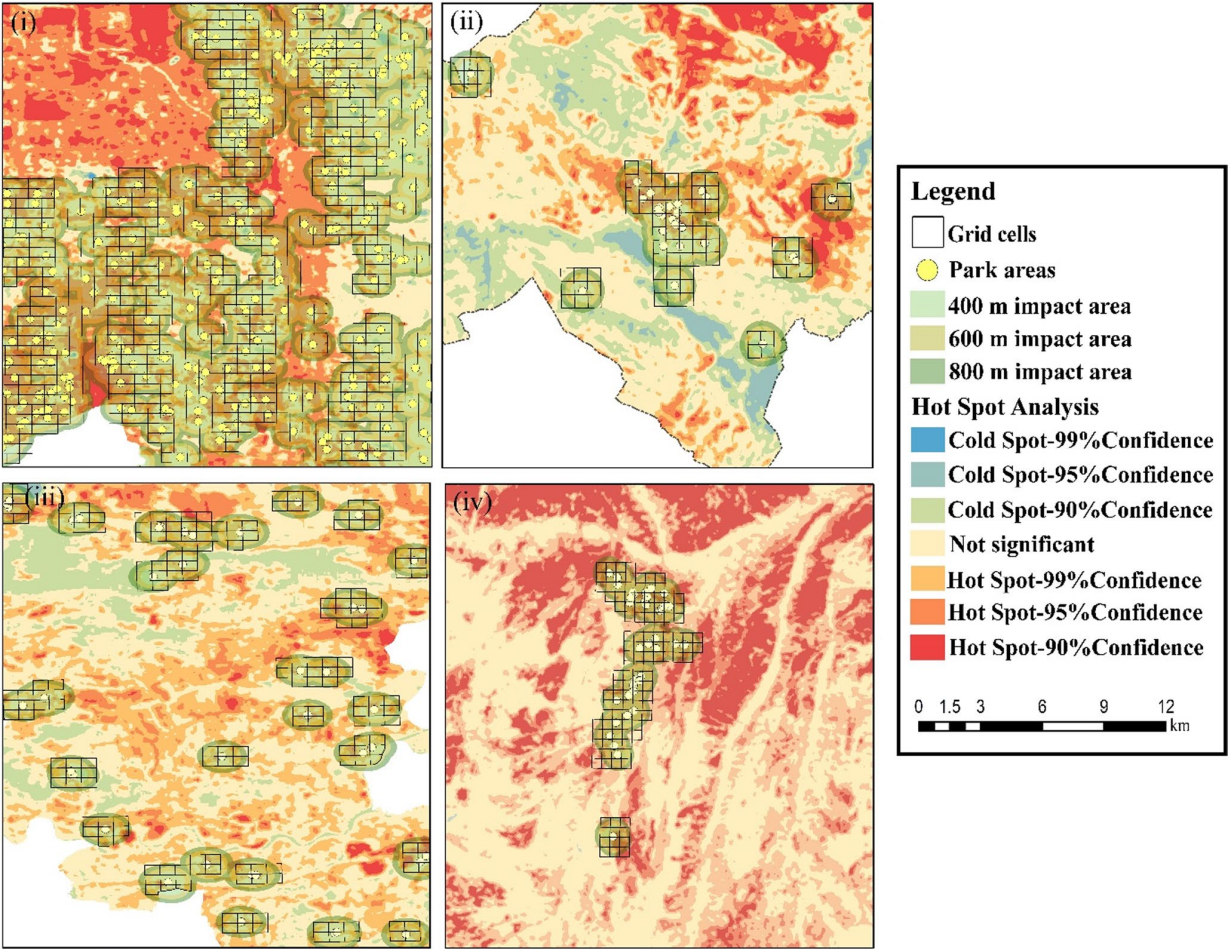
LST and cold–hotspot distribution maps (i: Salt Lake City, ii: L’Aquila, iii: Krakow, iv: Kastamonu)

The Getis-Ord (Gi*) analysis revealed a statistically significant spatial clustering of surface temperatures in the study area. As shown in Fig. 7, cold spot clusters, which reach a 99% confidence level, are particularly concentrated in the immediate vicinity of parks and within the 400-m buffer zone. This finding indicates that cells containing parks and neighboring areas have statistically significantly lower surface temperatures. This clustering of cold spots confirms that parks have a strong cooling effect, and this effect is not limited to the park area but extends to its surroundings.

Across the four study cities, the magnitude of LST reduction observed within the 400 m buffer zones surrounding UGS varied notably. Based on spatial regression outputs and LCZ-based LST distributions, Salt Lake City exhibited the strongest cooling response, with a maximum estimated LST reduction of approximately 4.2 °C. This was followed by Kastamonu (≈ 3.5 °C) and Kraków (≈ 3.1 °C), while L’Aquila showed comparatively more moderate cooling effects (≈ 2.8 °C). These values represent context-dependent cooling magnitudes, derived from spatially explicit analyses, rather than universal or threshold-based effects. Accordingly, inter-city differences are reported here descriptively, with their underlying drivers further interpreted in the Discussion section.

It is worth noting that the analyses presented in this study examine the relationship between LST and λ types within the 400 m impact zones of UGS. Within this spatial context, a stronger cooling response was observed in certain LCZs, particularly LCZ5 in Salt Lake City- where relatively higher levels of surrounding green presence were associated with lower LST values. However, the study does not explicitly quantify UGS coverage ratios within individual LCZs, nor does it aim to identify critical or universal threshold values of vegetation density associated with enhanced cooling effects. Accordingly, the results should be interpreted as reflecting relative and context-dependent thermal responses of different LCZs in proximity to parks, rather than definitive cooling thresholds based on vegetation percentage.

## Discussion

### Cooling efficacy of UGS across LCZs

The comprehensive analysis of UGS cooling effects across λs revealed several critical patterns that advance our understanding of urban heat mitigation strategies. In Salt Lake City, characterized by extensive dense urban LCZs (particularly LCZ5 [open high-rise] and LCZ6 [open low-rise]), UGS demonstrated remarkable cooling potential, with maximum LST reductions of 4.2 °C within 400-meter buffer zones (Adj. R² = 0.91, GWR model). This pronounced effect can be attributed to several synergistic factors: the high thermal storage capacity of the prevalent impervious surfaces in these zones creates strong temperature contrasts with adjacent vegetated areas, amplifying the observable cooling effect (Wei et al. 2025); the canyon geometry of LCZ5 and LCZ6 enhances airflow patterns that distribute cooler air from UGS into surrounding built environments; and the relatively arid climate of Salt Lake City increases the efficiency of evapotranspiration cooling from irrigated urban vegetation (Sarfo et al. 2023). The findings from L’Aquila presented an interesting counterpoint, where the cooling magnitude was more moderate (median LST reduction of 2.8 °C) despite substantial green space coverage. This apparent paradox can be explained by the city’s dominant natural λs (LCZA [dense trees] and LCZB [scattered trees]), which create a baseline cooling effect that reduces the marginal benefit of additional UGS. This phenomenon aligns with the principle of diminishing returns in ecosystem services (Song et al. 2025), where the thermal benefits of vegetation follow a nonlinear relationship with cover percentage. The pre-existing vegetative cover in L’Aquila’s natural LCZs maintains lower ambient temperatures, thereby reducing the temperature differential that can be achieved through additional greening interventions.

The intermediate results observed in Krakow (a 3.1 °C reduction) and Kastamonu (a 3.5 °C reduction) illustrate the complex interplay between urban form and the efficacy of green infrastructure in mixed-use landscapes. Krakow’s urban fabric, characterized by LCZ3 (compact low-rise) interspersed with agricultural areas (LCZD [low plants]), demonstrates how heterogeneous land cover creates microclimatic variability that affects UGS performance. The slightly stronger cooling in Kastamonu may reflect its unique urban forest configuration, where built areas (LCZ6) are embedded within extensive forest cover (LCZA), creating a more continuous cooling network. This supports the “green network” hypothesis proposed by Connors et al. (2013), which posits that interconnected vegetated systems provide more substantial thermal regulation than isolated green spaces. Yan et al. (2025) demonstrated the impact of vegetation loss and heat emissions in China’s major cities.

Notably, the cooling efficiency varied not only between, but also within, λ categories. In Salt Lake City’s LCZ5, areas with higher UGS coverage exhibited a relatively stronger cooling response compared to zones with lower vegetation density. Specifically, buffers with ≥ 30% UGS coverage showed approximately 18% greater LST reduction than those with 10–20% coverage (*P* < 0.05). Rather than representing a universal or statistically derived threshold, this pattern reflects a city- and LCZ-specific response under arid climatic conditions and open, high-rise morphology. No comparable vegetation coverage breakpoints were consistently observed across the other three cities, highlighting the context-dependent nature of UGS–LST relationships. These findings suggest that cooling benefits may follow nonlinear trends within certain urban forms, but further studies employing explicit threshold-detection methods are required to identify robust, LCZ-specific vegetation density limits. This threshold effect has important planning implications, suggesting that UGS design should prioritize achieving critical vegetation density thresholds rather than simply increasing the total green space area. These findings corroborate recent work by Yu et al. (2018) on the nonlinear relationship between vegetation cover and the magnitude of cooling. The study also revealed temporal dynamics in cooling performance, with peak effectiveness occurring in the early afternoon hours (13:00–15:00 local time), when solar radiation is at its strongest. This timing coincides with periods of maximum heat stress vulnerability, making UGS particularly valuable for public health protection during heatwaves (He et al. 2022). However, the nocturnal cooling persistence varied significantly by λ, with dense urban zones maintaining 1.2–1.8 °C of cooling through the night, while natural LCZs showed negligible nighttime effects. This diurnal pattern stresses the importance of considering daytime and nighttime thermal regimes in urban heat mitigation planning (Azhdari et al. 2018).

The hot spot analysis highlights the spatial extent of UGS cooling beyond the boundaries of individual parks. The statistically significant clustering of cold spots around parks, particularly within a 400-meter buffer, indicates that green spaces not only reduce local surface temperatures but also exert a measurable influence on their immediate surroundings. This finding reinforces the concept that the cooling potential of UGS is spatially diffuse and interacts closely with the surrounding urban morphology, emphasizing the importance of strategically locating green spaces to maximize their thermal regulation benefits (Shao and Kim 2022; Zhang et al. 2024). In other words, the cooling influence of UGS declines with increasing distance from the park, exhibiting the strongest effect within the immediate vicinity and gradually diminishing in more distant buffer zones (Liao et al. 2021; Zhu et al. 2021; Zhang et al. 2024). Conversely, hot spot clusters were predominantly found in densely built-up areas with high impervious surface coverage, illustrating the spatial concentration of UHIs where natural cooling is limited (Tanoori et al. 2024). The contrast between hot and cold clusters underscores the role of urban form in modulating UGS effectiveness: areas with low vegetation cover and high building density experience the most intense heat accumulation, whereas natural λ types or vegetated areas maintain lower surface temperatures. These results support a more targeted, morphology-sensitive approach to urban heat mitigation, where hot spot mapping can guide planners in prioritizing green interventions to achieve both local and neighborhood-scale cooling.

### Spatial autocorrelation and scale dependence

The spatial analysis revealed profound insights into how urban form influences the distribution and effectiveness of green space cooling. Salt Lake City’s exceptionally high spatial autocorrelation (I = 0.789) reflects its planned gridiron street network and homogeneous land use patterns, creating large contiguous zones with similar thermal properties. This finding aligns with the “urban climate archipelago” concept proposed by Stewart and Oke (2012), where cities comprise distinct thermal islands with sharp boundaries. The spatial consistency in Salt Lake City’s thermal patterns suggests that cooling interventions could be strategically implemented on a sector-wide basis rather than in isolated pockets. Krakow’s comparatively lower Moran’s I value (0.569) reveals a more complex thermal landscape shaped by its historical urban morphology and surrounding agricultural lands. The interspersed croplands (LCZD) create what we term a “thermal patchwork effect,” where alternating cool and warm zones disrupt spatial autocorrelation. This phenomenon was particularly evident in transition areas between the medieval city core (LCZ3) and suburban farmlands, where LST differences of up to 5.2 °C occurred within 500-meter distances. Such fragmentation presents both challenges and opportunities for heat mitigation. While it complicates city-wide cooling strategies, it also creates natural “cool corridors” that could be enhanced through targeted green infrastructure.

The GWR models provided unprecedented resolution in understanding scale effects, particularly the 400-meter threshold for meaningful UGS cooling. Our analysis revealed that this distance represents a critical inflection point where the cooling effect decays to < 50% of its maximum value, the cost-benefit ratio for green infrastructure begins to decline sharply, and microclimatic influences from the surrounding built environment dominate.

The logarithmic decay pattern (R² = 0.91 for the distance-cooling relationship) confirms theoretical models of urban boundary layer interactions. Within LCZ3 (compact low-rise), we observed extreme distance sensitivity - while a 10% increase in UGS cover yielded 0.154 °C cooling at 400 m, this dropped to just 0.042 °C at 600 m (*p* < 0.01). This nonlinear relationship suggests that urban planners should prioritize creating UGS networks with < 400 m spacing, implementing “cooling overlap zones” where green space influence areas intersect, and focusing on linear green elements (tree-lined streets, greenways) to maximize coverage within critical distance thresholds.

The scale analysis also revealed important interactions between UGS size and distance effects. Large parks (> 5 ha) maintained measurable cooling (0.8–1.2 °C) up to 800 m downwind, whereas small green spaces (< 1 ha) showed a limited influence beyond 200 m. Analysis supports the “hierarchical cooling” model proposed by Yu et al. (2018), which suggests that cities require both large “cool anchors” and distributed small green spaces for comprehensive heat mitigation. Notably, the 400 m effectiveness range varied by λ type: 450 m in LCZ5/6 (open high/low-rise), 350 m in LCZ3 (compact low-rise), and 300 m in LCZ2 (compact mid-rise). These differences likely reflect variations in urban canyon geometry and wind patterns that influence the dispersion of cool air. The findings strongly support context-sensitive green space planning considering the absolute distance and intervening urban morphology.

### Role of urban morphology in modulating cooling effects

The study’s findings reveal that urban structural characteristics play a pivotal role in determining the cooling efficacy of UGS. In Salt Lake City’s open high-rise zones (LCZ5), the reduced cooling performance (GWR coefficient = 0.274) compared to open low-rise areas (LCZ6; coefficient = 0.297) can be attributed to several interrelated factors. The vertical nature of high-rise buildings creates wind shadow effects that impede the horizontal dispersion of cool air from vegetated areas while trapping longwave radiation between structures. This phenomenon results in microclimates where ground-level temperatures remain elevated, despite the presence of UGS. Furthermore, the deep shading from tall buildings limits solar access for vegetation, reducing evapotranspiration rates by an estimated 15–25% compared to more open urban forms. These findings underscore the challenges of implementing conventional green space strategies in high-rise urban environments.

In contrast, Kastamonu’s scattered tree zones (LCZB) demonstrated superior cooling performance (coefficient = −0.238), highlighting the advantages of natural vegetation configurations. The multi-layered canopy structure of these areas provides optimal shade distribution while maintaining adequate air circulation. Additionally, the undisturbed ground cover in these natural zones retains higher soil moisture levels, which significantly enhances the evaporative cooling potential. The heterogeneous surface characteristics of these areas, combining tree canopies with bare ground, create an ideal albedo balance that minimizes heat absorption while maximizing cooling efficiency. These results emphasize the importance of preserving and incorporating natural vegetation patterns into urban planning.

The study’s results provide empirical evidence supporting context-sensitive approaches to urban heat mitigation, emphasizing that planners and designers should consider not only the quantity of green spaces but also their strategic placement and integration with urban form characteristics. Particularly in dense urban areas, the cooling benefits of UGS can be significantly enhanced by carefully aligning with built environment features and attending to microclimatic conditions. Building on this, the study advances urban climate research by applying a multi-city, LCZ-based comparative approach to evaluate UGS cooling effects across four mid-latitude cities Salt Lake City (USA), L’Aquila (Italy), Krakow (Poland), and Kastamonu (Türkiye) that share similar climatic and latitudinal contexts but exhibit distinct urban morphologies. By integrating MODIS-derived LST, λ mapping, hot spot analysis (Getis-Ord (Gi*)), and spatially explicit regression models (OLS and GWR), the study disentangles the relative influence of local morphology, green space configuration, and the spatial clustering of heat and cold spots on urban cooling. This approach not only highlights transferable patterns and context specific variations but also provides actionable insights for climate-responsive urban design in an era of increasing temperatures and urban heat challenges.

From an urban planning perspective, the results highlight the importance of considering LCZ-specific responses to the cooling influence of UGS, rather than relying on uniform greening strategies. At the same time, the present study does not identify critical vegetation coverage thresholds within individual λs; the observed variability in LCZ–LST relationships across cities suggests that the cooling efficiency of green infrastructure is likely to be context-dependent. In dense built environments, such as mid- and high-rise buildings, incremental increases in vegetation may not yield linear cooling benefits, indicating the potential existence of nonlinear or threshold-based responses. Future studies combining LCZ classification with quantitative vegetation metrics (e.g., green space coverage or canopy density) are therefore needed to identify LCZ-specific vegetation thresholds that can inform more targeted and effective urban heat mitigation strategies.

## Conclusion

This study confirms that UGS effectively reduce LST, with their cooling impact strongly influenced by local urban form and land use. The comparative, multi-city LCZ-based approach applied here highlights patterns and variations that are transferable across cities with similar climates but distinct morphologies. Key findings indicate that dense urban areas benefit most from targeted green interventions, natural zones provide a consistent baseline cooling effect, and the spatial configuration of UGS, particularly within a 400-meter radius, is critical for maximizing thermal relief. These results emphasize the importance of context-sensitive planning that integrates UGS design with specific LCZ characteristics, supporting resilient and livable urban environments in the face of rising temperatures.

### Implications for climate-adaptive urban planning

The findings of this study provide clear, data-driven guidance for urban planners and policymakers aiming to design climate-resilient cities. The use of advanced statistical models—such as Geographically Weighted Regression (GWR) with Akaike Information Criterion (AICc) values ranging from 1327 to 2871—underscores the reliability of the evidence presented. Three main strategies emerge for optimizing the placement and impact of UGS in mitigating LST.

### Prioritize green interventions in compact urban zones (LCZ3)

Among all LCZs, LCZ3 (compact low-rise) areas demonstrated the highest sensitivity to green space interventions. For instance, in Krakow’s LCZ3, a unit increase in green coverage led to a 0.214 °C reduction in LST. These densely built areas, often characterized by limited ventilation and high thermal mass, are especially vulnerable to heat buildup. By prioritizing UGS in these neighborhoods—through street trees, small parks, or green corridors—cities can achieve disproportionately high cooling benefits, improving thermal comfort and reducing public health risks. Even small increases in vegetation cover can make a significant difference, especially in limited spaces.

### Preserve and protect natural LCZs as climate buffers

Natural zones such as LCZD (low vegetation) and LCZB (scattered trees) act as critical regional coolers, even if their individual cooling coefficients seem modest (e.g., L’Aquila, LCZD = − 0.004). These areas provide a consistent baseline cooling effect and help regulate surrounding urban temperatures. Urban expansion into these natural LCZs risks eliminating their cooling services and worsening the UHI effect. Urban policy should therefore prioritize their conservation through tools such as zoning laws, green belts, urban growth boundaries, and land-use regulations. Preserving these spaces is as vital as creating new green areas.

### Apply the 400-meter buffer principle in planning

A key spatial insight from the study is that the cooling effect of UGS is strongest within a 400-meter radius. This aligns with the “5-minute walkability” standard often used in urban design and supports the creation of equitable, heat-resilient neighborhoods. Beyond this 400-meter threshold, the cooling effect declines sharply, with diminished returns beyond 600 m. To ensure consistent thermal relief and green space access, cities should implement a distributed network of UGS, ideally spaced 800 m apart to allow for overlapping cooling zones. This strategy promotes not only climate adaptation but also walkable, healthier, and more inclusive communities.

While previous studies have revealed the existence of cooling buffers around UGS, this study contributes to the literature by demonstrating that the magnitude and spatial consistency of these cooling effects systematically vary across different LCZs and urban contexts. Rather than treating buffer distance as a uniform planning rule, the study highlights how LCZ-specific morphology, urban density, and regional climatic conditions shape the effectiveness of the 400 m cooling zone. By integrating LCZ classification with spatial regression analysis across four climatically and morphologically distinct cities, this study provides a transferable yet contextually sensitive framework that surpasses distance-based generalizations and supports sensitivity-oriented, climate-adaptive urban planning.

### A framework for precision planning using LCZ mapping

Looking ahead, integrating LCZ-based urban zoning with green infrastructure planning offers a robust framework for climate-adaptive urban development. Combining high-resolution thermal data (e.g., MODIS LST) with LCZ maps enables planners to pinpoint high-risk zones and tailor interventions accordingly. For example, High-rise zones (LCZ5), where space is limited and shading is uneven, may benefit most from vertical greening systems, green roofs, or facade vegetation. Low-rise areas (LCZ6) are better suited to traditional parks, tree-lined streets, and community gardens. In both cases, complementary strategies such as reflective surfaces, permeable pavements, and water-sensitive urban design can amplify the cooling impact of green infrastructure.

### Limitations of the study and future directions

While this study provides valuable insights into the cooling effects of UGS across different Local LCZs, some limitations should be acknowledged. First, the reliance on MODIS data for LST introduced a resolution constraint (1 km), which may have overlooked micro-scale thermal variations within urban canyons or small green spaces (Li et al. 2018). Future studies could incorporate higher-resolution thermal data from Landsat or Sentinel-3 to capture finer details. Second, the analysis focused on summer months, potentially missing seasonal dynamics in UGS cooling efficiency; year-round monitoring would better inform climate adaptation strategies. Third, the study did not account for variations in vegetation types (e.g., grass vs. trees) or irrigation practices, which can significantly influence evapotranspiration rates (Wang et al. 2024). Future work should integrate field measurements of plant species and soil moisture to refine estimates of cooling. Lastly, the 400 m buffer for UGS impact was empirically chosen; a sensitivity analysis with varying distances (e.g., 200–800 m) could optimize planning guidelines.

Another limitation of this study is that the selected cities vary significantly in total area and population size, which naturally affects the number and spatial distribution of LCZ classes identified within each city. These differences may influence the relative representation of urban and natural LCZs and thus partially shape the observed relationships between LCZ types, UGS, and LSTs. However, the comparative framework of this research focused on cities located in similar mid-latitude climate zones but with contrasting urban morphologies, rather than cities of equal size or population. This design aims to isolate the role of local urban form in moderating cooling effects, regardless of city scale. However, future studies could address this limitation by including a larger sample of cities that represent a broader and more balanced range of sizes, population densities, and spatial extents. Such an approach would enable statistical generalization and a deeper understanding of how city-scale characteristics interact with LCZ-based cooling dynamics.

This study offers a practical roadmap for evidence-based, scalable heat mitigation. By focusing on: high-impact zones (e.g., LCZ3), preserving existing natural buffers, adopting the 400-meter planning threshold, and tailoring UGS types to LCZ characteristics, cities can significantly improve their climate resilience, thermal comfort, and equity outcomes. Future research should investigate synergies between UGS and other adaptation measures, such as cool roofs, urban water features, and community-based greening initiatives, to develop comprehensive, integrated strategies for urban climate adaptation. Moreover, future research directions should prioritize three areas: coupling LCZ-based models with climate projections to assess UGS efficacy under future warming scenarios (Zhou et al. 2017); evaluating socioeconomic barriers to UGS implementation in high-heat LCZs (e.g., LCZ5–LCZ6), where land costs may limit interventions (Kabisch et al. 2016); and exploring hybrid solutions (e.g., green roofs combined with reflective pavements) to amplify cooling in space-constrained urban cores (Sailor et al. 2012). Effectively addressing these problems involves implementing urban cooling and temperature reduction methods through ecosystem regulatory services, where the strategic use of green urban infrastructure becomes paramount.

In this study, the analysis focused on the 400-meter buffer zones surrounding urban parks in four cities, based on the assumption that this distance reflects the general walking accessibility and potential influence zone of parks on surrounding urban temperature. While the presence and spatial distribution of park areas were analyzed, variations in their ecological structure and design elements, which may significantly influence their cooling potential, were not included in the scope of this research due to data limitations. Future studies could benefit from incorporating detailed vegetation indices (e.g., NDVI, tree height, or canopy density) and morphological features of parks to understand better the relationship between park characteristics and their influence on urban thermal environments.

## Supplementary Information

Below is the link to the electronic supplementary material.

Supplementary Material

## Data Availability

The data that support the findings of this study are available from the corresponding author, upon reasonable request.
