# Supplementary material for: Cooling effect of urban green spaces: LCZ-based assessment comparing four cities at similar latitudes via hotspot and regression models
Source: Int J Biometeorol. 2026 Mar 19;70(4):95. doi: 10.1007/s00484-026-03172-x (PMC13002684; doi:10.1007/s00484-026-03172-x)
Supplement: Supplementary file 1 — Supplementary Material [file 484_2026_3172_MOESM1_ESM.docx]

**Supplementary for**

**Cooling Effect of Urban Green Spaces: LCZ-Based Assessment Comparing Four Cities at Similar Latitudes via Hotspot and Regression Models**

Dilara Yilmaz^1^, Oznur Isinkaralar^2,*^, Kaan Isinkaralar^3^, Emmanuel Yeboah^4^, Isaac Sarfo^5^, Ayyoob Sharifi^6^, Sevgi Öztürk^2^, Collins Oduro^7,8^, Ali Soltani^9,10^, Mohsen Roohani Qadikolaei^11^

^1^Department of Landscape Architecture, Graduate School of Natural and Applied Sciences, Kastamonu University, 37150, Kastamonu, Türkiye

Email: dlaraa.ylmaz@gmail.com

^2^Department of Landscape Architecture, Faculty of Engineering and Architecture, Kastamonu University, 37150, Kastamonu, Türkiye

Email: obulan@kastamonu.edu.tr

Email: sozturk@kastamonu.edu.tr

^3^Department of Environmental Engineering, Faculty of Engineering and Architecture, Kastamonu University, 37150, Kastamonu, Türkiye

Email: kisinkaralar@kastamonu.edu.tr

^4^School of Remote Sensing and Geomatics Engineering, Nanjing University of Information Science and Technology, 210044 Nanjing, Jiangsu, China.

Email: emmanuelyeboah@nuist.edu.cn (ORCID ID: 0000-0003-3838-6837)

^5^College of Geography and Environmental Science, Henan University, Kaifeng, Henan Province, China. Email: isaacsarfo@henu.edu.cn (ORCID ID: 0000-0002-6914-5764)

^6^The IDEC Institute and Network for Education and Research on Peace and Sustainability (NERPS), Hiroshima University, 739-8529, Higashi-Hiroshima, Japan,

Email: sharifi@hiroshima-u.ac.jp

^7^Department of Geography and Spatial Information Techniques, Ningbo University, Ningbo, 315211, China

^8^Ningbo Universities Collaborative Innovation Center for Land and Marine Spatial Utilization and Governance Research at Ningbo University, Ningbo, 315211, China

Email: collins@nbu.edu.cn

^9^School of Humanities, Arts and Social Sciences (HASS), University of New England, NSW, Australia

^10^City Futures Research Centre, University of New South Wales, Sydney, NSW, Australia

Email: a.soltani@unsw.edu.au

^11^Department of Urban Planning, University of Guilan, Rasht, Iran

Email: mroohani@phd.guilan.ac.ir

*Corresponding author Email: obulan@kastamonu.edu.tr

**Text**

**Retrieval of Land Surface Temperature (LST) from the MODIS data**

To match the spatial resolution of the LCZ map, MODIS LST data were resampled to 100*100 m, ensuring alignment with the LCZ classification. The “project raster” tool in the ArcGIS 10.4.1 program was used to adjust the map's resolution. The resampled MODIS LST values were also validated against Landsat-derived LST for selected dates, confirming their accuracy. The accuracy of the resampled MODIS LST was further confirmed through comparison with Landsat-derived LST. To assess the agreement between MODIS and Landsat-derived LST, Pearson correlation analyses were conducted for each city. The correlation coefficients (R²) and RMSE values were as follows: Salt Lake City, R² = 0.8405, RMSE = 0.59; L’Aquila, R² = 0.7970, RMSE = 0.57; Krakow, R² = 0.8180, RMSE = 0.60; and Kastamonu, R² = 0.8988, RMSE = 0.61. These results indicate a strong positive relationship between MODIS and Landsat LST across all four cities, confirming the reliability of MODIS-derived summer LST for subsequent analyses**.** This approach provided both temporal representativeness of summer conditions and spatial consistency with the morphological data used in the analysis.

**Statistical relationship between UGS and LST in LCZs**

The Ordinary Least Squares (OLS) model in ArcGIS was used to define the relationship between LST during the summer months and LCZs within the 400 m impact areas of parks. The adjusted R² coefficients for the OLS regression models are 0.714 for Salt Lake City, 0.546 for L’Aquila, 0.394 for Kastamonu, and 0.331 for Krakow. These values indicate a strong relationship in Salt Lake City, a moderate relationship in L’Aquila, and weaker relationships in Kastamonu and Krakow. Although the strength of association varies by city, the results consistently suggest that LCZs are relevant predictors of LST to varying degrees. Statistical significance of the models was also confirmed through associated p-values **(Appendix Table S3)**. To ensure the validity of the regression models, multicollinearity among the LCZ variables was assessed using the Variance Inflation Factor (VIF). VIF is commonly used to detect the degree of interdependence among explanatory variables in a regression model. VIF was calculated separately for each city, based on the LCZ variables included in that city’s model, to test the independence of predictors within each local dataset rather than across cities. A VIF value above 10 is generally considered to indicate problematic multicollinearity. In this study, all VIF values were well below this threshold, with the highest value being 2.243 in Salt Lake City and the lowest 0.124 in Krakow, confirming that there is no significant multicollinearity among LCZ variables within the 400 m buffer areas. This ensures that the estimated coefficients for LCZs reliably capture their individual contributions to explaining spatial variations in LST.

Then, Global Moran’s I in ArcGIS was used to test the spatial autocorrelation between the LCZs in the 400 m impact area of the parks and the summer LST values in **Appendix Table S4**. The Global Moran’s I results are given that the Moran I value for LST of the OLS models of the cities is strongly significant at the 0.001 level and has positive z-score values defining high spatial autocorrelation. Therefore, the GWR model was used in the study.

**Appendix Table S5** shows the results of the GWR model. R² and Adj. R² values are above 0.50. Akaike Information Criterion (AICc) values are between 1327.498 and 2871.897. These values show that the GWR model is suitable for use in the study. Suppose the effect of the spatial pattern of the LCZ on LST is ranked from high to low by Adj. R², Salt Lake City (0.91) is in first place, followed by L’Aquila (0.80), Kastamonu (0.49), and Krakow (0.47).

When the GWR model results are examined in terms of coefficients, it is seen that all coefficients in urban areas (LCZ1-LCZ9) have a positive relationship with LST. At the same time, the highest Adj. The R² and R² coefficients are found in Salt Lake City (Adj. R²: 0.91, R²: 0.93) and L’Aquila (Adj. R²: 0.80, R²: 0.81), indicating that the LST effect is more intense in LCZs in these cities, while at the same time, the impact of LCZs located within 400 m of the parks on LST is also strong.

In general, built types have a positive relationship with LST, while natural areas have a negative relationship with LST. In Salt Lake City, where urban areas are dense, the positive coefficients of LCZ5 (0.274) and LCZ6 (0.297) indicate that the increase in these areas will increase the LST values in the summer months. In L’Aquila, which stands out with its natural areas, the negative coefficients of LCZA (-0.124), LCZB (-0.198), and LCZD (-0.004) indicate that the increase in these areas will decrease the LST values in the summer months. Similar situations are observed in the cities of Krakow and Kastamonu. The results show that there is a positive relationship between urban LCZs in the 400 m impact area of park areas and LST. At the same time, there is a negative relationship between natural LCZs and LST. The spatial distribution of LCZ types significantly impacts the differentiation of these impact levels in four different cities.

**Cooling Effects of Parks Identified through Hot Spot Analysis**

Hot spot areas, indicated in red on the map, are concentrated in regions without parks, where impervious surfaces are prevalent and building density is high. This demonstrates that the urban heat island effect is most intense in these areas and that cooling capacity is insufficient. High temperature clusters, particularly in the northern and northeastern regions, indicate critical areas with weak green infrastructure. Furthermore, it was determined that hot spot clusters largely occur in areas with dense artificial surfaces and structures within the LCZ classification, while in areas with natural LCZ types (forest, meadow, water surface, etc.), either cold spot clusters are observed or no statistically significant temperature difference is observed. This supports the fact that the morphological characteristics of the urban fabric play a decisive role in surface temperature distribution.

Buffer analyses showed that the cooling effect of parks decreases with distance. Cold spot density is highest within the 400 m buffer, while the effect gradually weakens in the 600 and 800 m buffer zones, but is still observable. This finding suggests that the cooling effect of parks exhibits a gradual decrease and has the strongest effect in the immediate vicinity. Thus, the microclimate moderating effect of parks is confirmed both statistically and spatially.

**Role of urban morphology in modulating cooling effects**

The regression analysis delineated the thermal behavior differences between built and natural environments. Urban LCZs consistently exhibited positive relationships with land surface temperature (LST), with compact low-rise areas (LCZ3) showing the most pronounced warming effect. This aligns with established urban climatology principles regarding the heat-retention properties of dense urban fabrics. Conversely, all-natural LCZ categories demonstrated significant cooling capacities, particularly dense tree zones (LCZA). The cooling efficiency of these natural areas followed a nonlinear trajectory, reaching maximum effectiveness at 60-70% vegetation cover before plateauing, suggesting an optimal threshold for green space design. Three key structural metrics emerged as critical moderators of UGS performance: sky view factor, street canyon aspect ratio, and surface material composition. Areas with restricted sky visibility (SVF <0.5) showed markedly reduced cooling benefits from UGS, while narrow street canyons (H/W >1.5) diminished green space effectiveness by up to 40%. These findings have important implications for urban design, suggesting that green infrastructure strategies must be tailored to specific urban morphologies. In high-rise districts, vertical greening systems may prove more effective than traditional ground-level parks, while low-rise areas benefit most from conventional green space development. For compact urban forms, integrated solutions combining vegetation with reflective materials and carefully oriented ventilation corridors appear most promising for heat mitigation.

**Table S1.** Sample area LULC distribution

|  | **LULC distribution*** | | | | | | | | | |
| --- | --- | --- | --- | --- | --- | --- | --- | --- | --- | --- |
| **LULC** | **Salt Lake City** | | **L’Aquila** | | | **Krakow** | | **Kastamonu** | | |
|  | **Km²** | **%** | **Km²** | | **%** | **Km²** | **%** | **Km²** | | **%** |
| Built-up area | 487 | 85.41 | 155 | 27.19 | | 168 | 29.52 | 89 | 15.55 | |
| Crops | 11 | 1.93 | 104 | 18.32 | | 257 | 45.11 | 274 | 48.1 | |
| Rangeland | 56 | 9.82 | 165 | 28.97 | | 18 | 3.11 | 75 | 13.09 | |
| Forest |  |  | 115 | 20.24 | | 103 | 18.14 | 120 | 21.13 | |
| Water surfaces | 2 | 0.35 | 18 | 3.11 | | 23 | 4.12 | 3 | 0.59 | |
| Bare ground | 14 | 2.46 | 12 | 2.17 | |  |  | 9 | 1.54 | |
| *LULC distributions were calculated in ArcGIS program. | | | | | | | | | | |

**Table S2.** Validation of MODIS LST with Landsat derived LST

| **Cities** | **R²** | **RMSE** |
| --- | --- | --- |
| **Salt Lake City** | 0.8405 | 0.59 |
| **L’Aquila** | 0.7970 | 0.57 |
| **Krakow** | 0.8180 | 0.60 |
| **Kastamonu** | 0.8988 | 0.61 |
| *R²: Pearson correlation coefficient.  RMSE: Root mean square error P <0,005* | | |

**Table S3.** Adjust R² of OLS regression models

| **Cities** | **R²** | **Adj. R²** | **VIF** |
| --- | --- | --- | --- |
| Salt Lake City | 0.731 | 0.714 | 2.243 |
| L’Aquila | 0.597 | 0.546 | 1.644 |
| Krakow | 0.364 | 0.331 | 1.124 |
| Kastamonu | 0.393 | 0.394 | 1.589 |
| ** represents p < 0.01 in the significance test | | | |

**Table S4.** Spatial autocorrelation results

| **Cities** | **Moran I** | **Z-Score** | **P-Value** |
| --- | --- | --- | --- |
| Salt Lake City | 0.789 | 37.347 | 0.000 |
| L’Aquila | 0.631 | 30.021 | 0.000 |
| Krakow | 0.569 | 32.957 | 0.000 |
| Kastamonu | 0.581 | 29.745 | 0.000 |

**Table S5.** Statistical description of GWR mean coefficient

|  | **GWR Coefficient** | | | |
| --- | --- | --- | --- | --- |
| **LCZs** | **Salt Lake City** | **L’Aquila** | **Krakow** | **Kastamonu** |
| LCZ1 | 0.082 |  |  |  |
| LCZ2 | 0.076 | 0.121 |  |  |
| LCZ3 | 0.154 | 0.206 | 0.214 | 0.012 |
| LCZ4 | 0.054 |  | 0.231 |  |
| LCZ5 | 0.274 | 0.183 | 0.198 | 0.021 |
| LCZ6 | 0.297 | 0.065 | 0.174 | 0.224 |
| LCZ8 | 0.164 | 0.011 | 0.016 | 0.105 |
| LCZ9 | 0.033 | 0.024 | 0.003 | 0.012 |
| LCZA | -0.062 | -0.124 | -0.098 | -0.214 |
| LCZB | -0.058 | -0.198 | -0.012 | -0.238 |
| LCZD | -0.045 | -0.004 | -0.031 | -0.254 |
| LCZE | -0.003 |  |  |  |
| LCZF | 0.004 |  |  | -0.005 |
| **AICc** | 2871.897 | 1327.498 | 1335.549 | 2345.858 |
| **R²** | 0.93 | 0.81 | 0.56 | 0.52 |
| **Adj. R²** | 0.91 | 0.80 | 0.47 | 0.49 |
|  | Adj.R2 < R2 | Adj.R2 < R2 | Adj.R2 < R2 | Adj.R2 < R2 |
| Constant parameter: Sample area mean LST values | | | | |


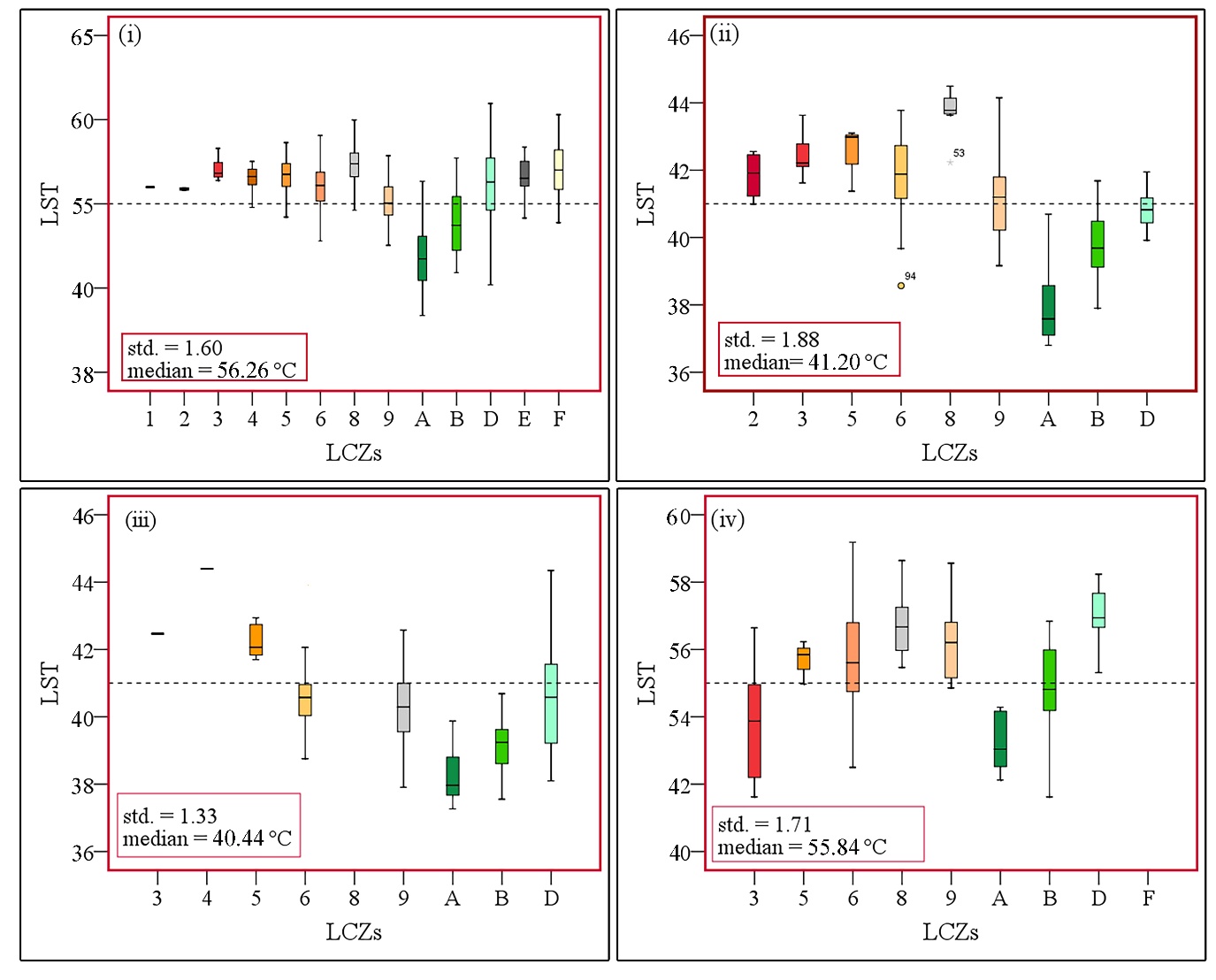


**Figure S1.** Study area average LST in LCZs within 400 m of UGS (i: Salt Lake City, ii: L’Aquila, iii: Krakow, iv: Kastamonu)
